# Protein Crystals Nucleated and Grown by Means of Porous Materials Display Improved X-ray Diffraction Quality

**DOI:** 10.3390/ijms231810676

**Published:** 2022-09-14

**Authors:** Christo N. Nanev, Emmanuel Saridakis, Lata Govada, Naomi E. Chayen

**Affiliations:** 1 Rostislaw Kaischew Institute of Physical Chemistry, Bulgarian Academy of Sciences, Acad. G. Bonchev Str. Bl. 11, 1113 Sofia, Bulgaria; 2 Structural and Supramolecular Chemistry Laboratory, Institute of Nanoscience and Nanotechnology, National Centre for Scientific Research “Demokritos”, 15310 Athens, Greece; 3 Section of Biomolecular Medicine, Division of Systems Medicine, Department of Metabolism, Digestion and Reproduction, Faculty of Medicine, Imperial College London, London W12 0NN, UK

**Keywords:** macromolecular crystallization, protein crystal nucleation, protein crystal growth, nucleation theory, nucleants, porous nucleation-inducing materials, bioglass

## Abstract

Well-diffracting protein crystals are indispensable for X-ray diffraction analysis, which is still the most powerful method for structure-function studies of biomolecules. A promising approach to growing such crystals is the use of porous nucleation-inducing materials. However, while protein crystal nucleation in pores has been thoroughly considered, little attention has been paid to the subsequent growth of crystals. Although the nucleation stage is decisive, it is the subsequent growth of crystals outside the pore that determines their diffraction quality. The molecular-scale mechanism of growth of protein crystals in and outside pores is theoretically considered. Due to the low degree of metastability, the crystals that emerge from the pores grow slowly, which is a prerequisite for better diffraction. This expectation has been corroborated by experiments carried out with several types of porous material, such as bioglass (“Naomi’s Nucleant”), buckypaper, porous gold and porous silicon. Protein crystals grown with the aid of bioglass and buckypaper yield significantly better diffraction quality compared with crystals grown conventionally. In all cases, visually superior crystals are usually obtained. Our theoretical conclusion is that heterogeneous nucleation of a crystal outside the pore is an exceptional case. Rather, the protein crystals nucleating inside the pores continue growing outside them.

## 1. Introduction

Providing a significantly higher resolution over a wider range of proteins, as compared to nuclear magnetic resonance and cryo-electron microscopy, X-ray diffraction studies account for 89% of structures deposited in the Protein Data Bank. Thus, even nowadays, X-ray diffraction continues to be a key method in structural biology. Unfortunately, however, protein crystals of sufficiently high diffraction quality are notoriously difficult to grow; this problem has been mitigated using porous materials [1,2,3,4,5,6,7,8,9,10,11]. Due to the synergistic effects of diffusion in confined pore spaces and protein adsorption on pore walls, a local concentration increase, which is just sufficient for crystal nucleation to occur, can be created in pores. Furthermore, excessively high supersaturation is highly unlikely to be achieved [12].

We have already considered the nucleation of protein crystals in pores, as can be seen in [13], because it makes the difference between the success and failure of any crystallization attempt. In doing so, we have shown that a prerequisite for growing 3D crystals in pores is to have already formed completely stable (i.e., destined to grow) 2D crystal nuclei, which are protein layers of monomolecular thickness. Such completely stable crystal nuclei require the cohesive energy Δ*G*_v_ (which maintains the integrity of a crystalline cluster) and the destructive energy Δ*G*_s_ (which tends to tear it up) to be equal. However, in contrast to the intensive theoretical study of protein crystal nucleation in pores, relatively little attention has been paid to the subsequent growth of the nucleated crystals. It was merely noted that “once nucleated, such crystallites continue their growth outside the pore orifice, forming 3D crystals.” [13].

In this paper, by considering the molecular-scale mechanism of growth of protein crystals that are initially inside pores and subsequently outside them, we show that the growth process is favored energetically in both cases. In other words, because under supersaturation conditions the crystal growth proceeds spontaneously, there is no need to form 3D nuclei—neither inside nor outside the pore. Most importantly, our consideration provides a plausible theoretical explanation of experimental observations (some of which are detailed below), which show that the use of various porous materials as nucleants contributes to the growth of protein crystals of a better diffraction quality.

## 2. Results 

### 2.1. Growth of Protein Crystals inside Pores

Firstly, and most importantly, there is a considerable difference between protein crystal growth in the confined pore space and growth in the open space outside the pore. Inside a pore, the growth of a crystal merely leads to an increase in volume, whereas the crystal surface, which is exposed to the destructive action of the water molecules, does not change in size (the reason being that the size of the pore hardly changes at a molecular distance). Hence, importantly, the destructive efficacy of the water molecules with respect to the protein crystal in the pore does not change. On the other hand, the growth of a crystal outside the pore leads to a simultaneous increase in both its volume and surface; indeed, the larger the crystal surface, the more intensive the destructive action of the water molecules.

The growth of a 3D protein crystal in a pore starts by attaching molecules to a previously formed, completely stable 2D crystal nucleus (for the latter, see Ref. [13]). The first protein molecules impinging on the nucleus are strongly and sufficiently connected, provided they are simultaneously bound to the pore wall and to the existing stable 2D nucleus. Then, additional molecules arrive, and they are bound even more strongly at kink sites formed by the first molecules and the lower crystal layer. The result of such a growth process is a complete protein layer of monomolecular thickness, deposited onto the pre-existing stable 2D crystal nucleus. When reiterated several times, this process leads to the transformation of the stable 2D crystal nucleus into a 3D protein crystal.

**Figure 1:**
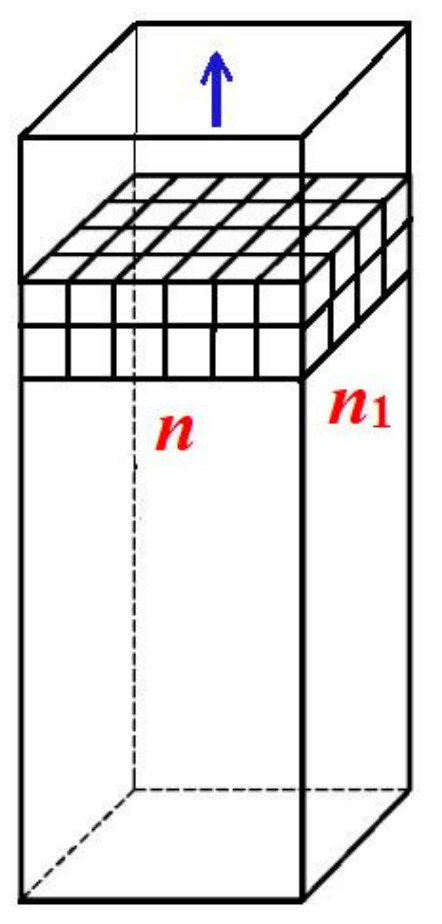
A prismatic pore with a Kossel crystal growing in it; two layers of monomolecular thickness are shown. The numbers of molecules at the edges of the Kossel crystal are denoted by *n* and *n*_1_. The growth direction of the crystal is shown by a blue arrow.

Since, as shown in Ref. [13], real non-regularly shaped pore orifices yield theoretical results that are very similar to those obtained with idealized pore shapes, we expect that the conclusions reached in this paper for regular pore shapes are generally valid. Therefore, we start the quantitative analysis of the formation of a 3D protein crystal in a pore by considering the simplest crystal lattice, the so-called Kossel crystal (a simple cubic symmetry crystal, constituted by small cubes held together by equal forces, as shown in Figure 1). Importantly, the formation of a 3D protein crystal from a pre-existing 2D completely stable nucleus inside the pore is an energetically driven process. The primary cause of this is the substantial increase in cohesive crystal energy Δ*G*_v_, resulting from the increased number of intermolecular bonds between the two protein layers of monomolecular thickness (whereas the crystal destructive energy Δ*G*_s_ remains the same). A stabilizing role is also played by the interaction of the protein molecules with the walls of the pore. Surrounded by four pore walls, the crystal inside it is substantially more stable than the 3D crystals formed (and growing) on foreign surfaces, as can be seen in [14]. However, the bonding of the protein molecules to the pore walls depends on the type of protein and porous material. All this makes the calculation of the balance between the crystal cohesive and destructive energies very difficult. To circumvent this problem, *only for the growth of protein crystals inside pores*, the bonding to pore walls (which is weaker than the bonding between the protein molecules in the crystal) is not considered. The price paid for this simplification is that the results are merely indicative.

Counting only the number of intermolecular bonds between the first and second crystal layers, we obtain *nn*_1_ bonds, where *n* and *n*_1_ denote the number of molecules at the edges of the Kossel crystal, as in Figure 1. To these, we must add the (*n* − 1)(*n*_1_ − 1) bonds that account for the intermolecular bonds within the layer (obviously the same as in the first layer of monomolecular thickness). As a result, the increase in the number of intermolecular bonds is: $\frac{\left[nn_{1}+\left(n-1\right)\left(n_{1}-1\right)\right]}{\left(n-1\right)\left(n_{1}-1\right)}=1+\frac{nn_{1}}{\left(n-1\right)\left(n_{1}-1\right)}>2$, i.e., the growing crystal becomes more than twice as strong.

**Figure 2:**
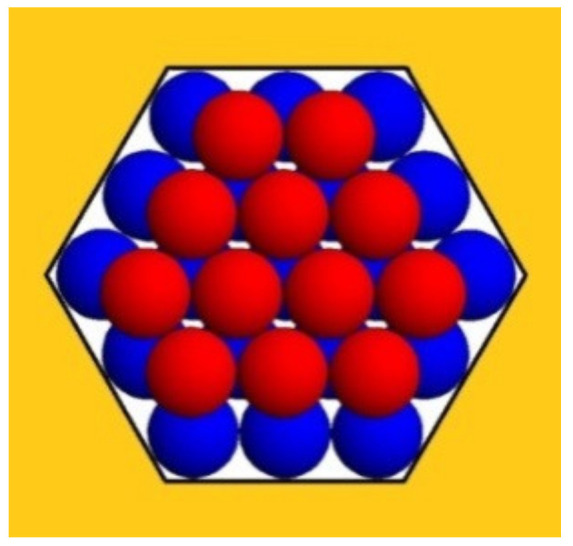
The two closest-packed layers of monomolecular thickness formed in a hexagonal pore orifice are shown in top view: the completely stable crystal nucleus with edge length *L* = 3 (layer A) is shown by blue balls and a second (ditrigonal) layer B (red balls) is deposited on it.

More generally, the increase in Δ*G*_v_ depends on the type of crystal lattice. Because the Kossel crystal has a relatively low-density packing, the increase in the number of intermolecular bonds in the Kossel crystal will be compared with that in the closest-packed crystal lattices (e.g., see Figure 2); we hope that this comparison sheds additional light on the spontaneous growth of protein crystals in pores. 

Figure 2 depicts a second monomolecular layer of a hexagonal close-packed (HCP) crystal that is deposited onto the completely stable nucleus (the blue balls). This is a ditrigonal layer, which is a little smaller than the first (hexagonal) layer. Therefore, it does not touch the pore walls (meaning there is no interaction between the protein molecules and the walls of the pore). However, each molecule in the ditrigonal layer (like in any close-packed crystal structure) interacts with three molecules underneath it—meaning that the energy needed for stripping-off one overlaying layer is three times larger. Consequently, this energy contribution overcompensates for the absence of interaction between protein molecules and pore walls (see Table 1 below).

The third layer in HCP crystals is also hexagonal; therefore, it touches the pore wall. However, most important is the fact that the destructive energy Δ*G*_s_, which tends to tear up the crystal, is always equal to the number of molecules in the uppermost (ditrigonal or hexagonal) crystal layers, which are exposed to the destructive action of the water molecules. (Evidently, only the molecules on the crystal surface interact with the water.).

The numbers of molecules and the numbers of intermolecular bonds in the closest-packed (ditrigonal and hexagonal) monolayers are known from crystallographic equations. Using these, we can perform numerical calculations of the relations between Δ*G*_v_ and Δ*G*_s_ for crystals with one, two, three etc., layers. In doing so, we denote by *λ* the number of molecules in the longer ditrigonal crystal edges (which equals the number of molecules in the hexagonal layer beneath it, as seen in Figure 2). As such, the number of molecules in the ditrigonal monolayers denoted by *Z* is:

(1) $$Z=3{\left(\lambda \;–\;1\right)}^{2}\;\;$$

Equation (1) gives *Z* = 12, 27, 48, 75... for *λ* = 3, 4, 5, 6..., respectively.

And the number *z* of molecules in hexagonal layers, the edge lengths of which are determined from the number *L* of molecules in them, is:

(2) z=3L(L−1)+1

Equation (2) gives *z* = 19, 37, 61, 91... for *L* = 3, 4, 5, 6..., respectively.

The number of bonds Δ*G*_v_^d^ in ditrigonal layers is given by the following equation:

(3) $$\Delta G_{v}^{d}=\left(3\lambda -5\right)\left(3\lambda -3\right)$$

where *λ* corresponds to the longer edge of the ditrigonal layer.

The number of bonds Δ*G*_v_^h^ in the hexagonal crystal layer is obtained from the crystallographic formula:

(4) $$\Delta G_{v}^{h}=\left(3\lambda -3\right)\left(3\lambda -2\right)$$

With $\psi_{d}$ being the destructive energy per bond, the destructive energies Δ*G*_s_ are, respectively:

(5) $$\Delta G_{s}^{d}=Z\psi_{d}$$

and:

(6) $$\Delta G_{s}^{h}=z\psi_{d}\;$$

The results of the calculations performed by means of these equations are shown in Table 1, where the bonding energy is denoted by $\psi_{b}$. Since, again, the interactions with the pore walls are not considered, these results are indicative only (and serve merely for comparison with the results of the Kossel crystals). A direct comparison shows that, in both cases, the growth of the protein crystals in pores is increasingly stimulated as the pores become wider. However, it is also evident that the wider the pore, the less important the positive effect of the capillary walls becomes. Thus, there is an optimal range of pore orifice widths.

The sharp increase in crystal stability with the thickening of the HCP crystal shown in Table 1, although somewhat mitigated for larger crystals, suggests that the growth of protein crystals in pores is, in general, an energetically driven process.

The fourth crystal layer, being again ditrigonal, has a smaller number of protein molecules exposed to the destructive action of the water molecules. This strengthens our conclusion that the growth of protein crystals in pores occurs by consecutive deposition of layers of monomolecular thickness and, being energetically favored, it is ceaseless. Therefore, there is no need to form a three-dimensional crystal nucleus to continue the growth of the protein crystal inside the pore.

Finally, it is worth noting that point defects (vacancies and interstitials) increase the conformational entropy and thus stabilize the crystals formed inside the pores [13]. On the other hand, such small crystals hardly tolerate the formation of dislocations; it is therefore more probable that dislocations arise in the crystals at later growth stages outside the pores.

### 2.2. Spontaneous Growth of the Protein Crystals outside the Pores

Because the volume of the protein crystal in the pore is negligible in comparison with the volume outside it, the latter growth stage is of prime importance for the diffraction ability of the protein crystal. Therefore, this stage of protein crystal growth deserves special attention. Importantly, when the protein crystal reaches the orifice of the pore, it starts growing at a protein concentration which is lower to that inside the pore: its growth is therefore slowed down. Additionally, the slower the crystal grows, the more perfect it is. The reason for this is that fewer impurities are incorporated by the slow-growing crystal, which leads to fewer defects in its lattice, meaning an improved diffraction quality [6]. Therefore, by lowering the supersaturation under which the crystals nucleate and grow (compared to the supersaturation needed for conventional crystal nucleation), the use of porous nucleants contributes to the further success of X-ray protein crystallography. Our experiments confirmed this theoretical suggestion [7].

**Figure 3:**
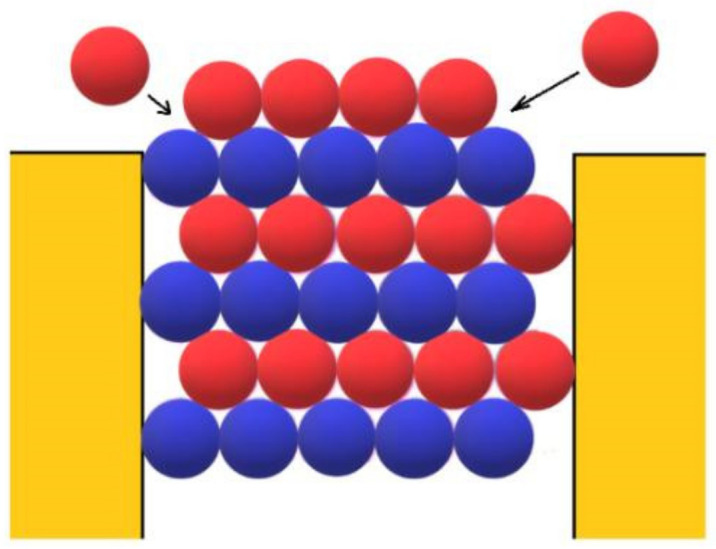
HCP crystal emerging from a pore having the shape of a rectangular prism (side view, as in Figure 1). The alternate layers are hexagonal A (blue balls) and ditrigonal B (red balls). Protein molecules are attached (at places shown by the arrows) to the crystal for growth outside the pore.

Let us consider the growth of protein crystals outside the pores in more detail. Firstly, the open space outside the pore is already advantageous for growing 3D protein crystals. As they are expanding laterally, those crystals are fed from more directions; thus, the effect of the reduced protein concentration outside the pore is somewhat mitigated. Secondly, because the binding energy between the two monomolecular protein layers is added to the binding energy between the molecules in the newly deposited monomolecular layer (see Figure 3), the binding energy Δ*G*_v_ is substantially increased. Therefore, we could suggest that the wider the pore orifice, the more energetically favored the growth of the crystal outside it. However, a very wide pore would approach the limit of a flat surface. On the other hand, since the pore opening is reached and the protein molecules enter the pore with the same probability with which they reach an equally large flat surface area, the narrower the pore orifices, the less accessible they are [12]. Additionally, the narrower the pore, i.e., the smaller the crystal nucleus, the higher the supersaturation required for its formation. As already mentioned, however, excessively high supersaturation can hardly result from the synergistic effects of diffusion in the confined pore spaces and protein adsorption on pore walls. Thus, we conclude again that there is an optimal range of pore orifice widths. Considering all the above arguments, we concluded that pores narrower than about 1 μm can accumulate enough protein to induce crystal nucleation [12].

To approach this issue quantitatively, we consider a crystal in a pore to have the same volume as a completely stable homogeneously formed crystal nucleus, which is expected to grow steadily [15,16]. The size of the latter is determined using the equilibration between the cohesive energy that maintains the integrity of a crystalline cluster and the destructive energy that tends to tear it up, i.e., the change in Gibbs free energy is ΔG=0 (recalling that the completely stable homogeneously formed crystal nucleus is 50% larger than the 3D critical crystal nucleus.).

**Figure 4:**
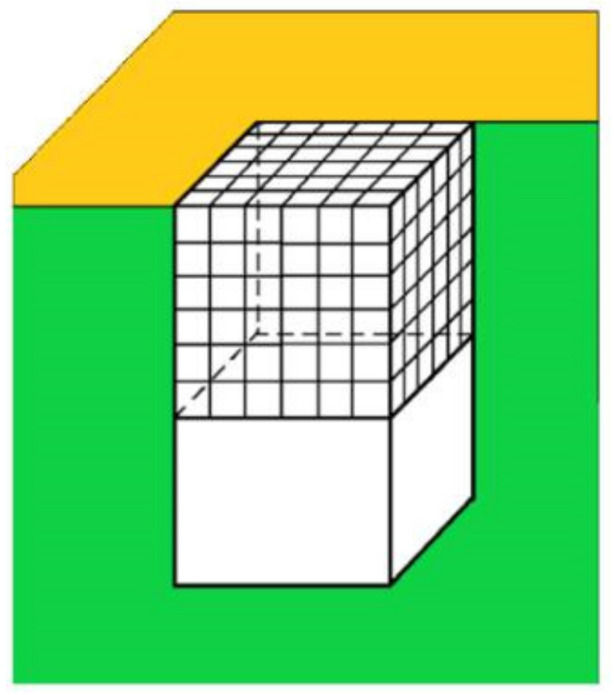
Cross-section (green) of a pore with a cubic crystal that just reaches the pore opening; the surface of the porous material is in gold. The protein crystal is in equilibrium with the solution trapped beneath it.

**Figure 5:**
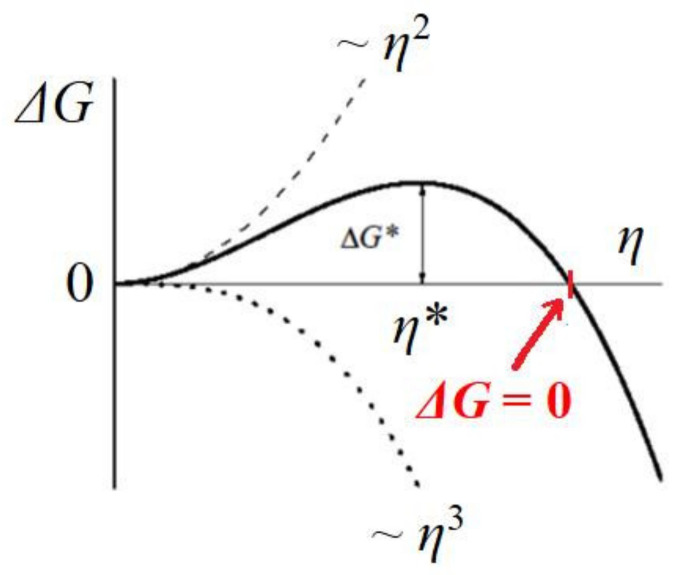
Change in Gibbs free energy Δ*G* with the number *η* of building blocks at the edge of the crystal. The critical nucleus size η* arises at ΔG*. The red arrow shows the point where ΔG=0.

Crystals in pores can have diverse shapes, but a calculation of the value of the supersaturation needed for the growth of the crystal outside the pore can be obtained by looking at a cubic crystal that has just reached the pore opening (see Figure 4). The change in Gibbs free energy Δ*G* (see Figure 5), which is required for crystal formation, is a sum of two terms: (1) the free energy gain resulting from the transfer of molecules from the supersaturated mother phase into the crystal (which is proportional to its volume, the so-called ‘volume term’); and (2) the free energy penalty, imposed due to the formation of the new interface (i.e., a ‘surface term’, which is proportional to the total area of the crystal). As such, we write the change in Δ*G* needed for the homogeneous formation of a cubic crystal constituted by *η* molecules:

(7) $$\Delta G=-\eta \Delta \mu +S\gamma_{c}=-\eta \Delta \mu +6\delta^{2}\eta^{2/3}\gamma_{c}$$

where *S* is the total surface of the nucleus, and *δ* the edge length of the crystal building block; *γ_c_* [erg/cm^2^] is the surface free energy.

For crystallization in solutions, the supersaturation $\Delta \mu =k_{B}Tln\left(\frac{c}{c_{e}}\right)$ is the driving energy for crystal nucleation; *k*_B_ is the Boltzmann constant; *T* is the absolute temperature; *c* is the actual concentration; and *c*_e_ is the equilibrium with respect to an “infinitely” large crystal (as usual, activity coefficients equal to 1 are assumed).

Thus, for a completely stable and homogeneously formed cubic crystal nucleus, the equilibration between cohesive energy Δ*G*_v_ and destructive energy Δ*G*_s_ (i.e., ΔG=0, see Figure 5) in Equation (7) gives:

(8) $$\eta \Delta \mu_{v\;}-6\delta^{2}\eta^{\frac{2}{3}}\gamma_{c}=0$$

where Δ*μ*_v_ denotes the supersaturation needed for homogeneous formation of the completely stable crystal nucleus.

Dividing Equation (8) by *η*, we obtain:

(9) $$\Delta \mu_{v\;}=6\delta^{2}\eta^{-1/3}\gamma_{c}$$

Of prime importance in our consideration is the fact that a large part of the surface of the crystal in the pore is protected from the destructive action of the water molecules—the latter can attack only a small part of its surface, the one that protrudes from the pore. Thus, the vulnerable surface of the crystal is reduced sixfold, and for the completely stable crystal face at the pore opening (at  ΔG=0), Equation (7) becomes:

(10) $$\Delta G=-\eta \Delta \mu_{p}+\delta^{2}\eta^{\frac{2}{3}}\gamma_{c}=0$$

where Δ*μ*_p_ is the supersaturation at which the cubic crystal shown in Figure 4 is expected to grow steadily.

Again, dividing Equation (10) by *η*, we obtain the value of this supersaturation:

(11) $$\Delta \mu_{p}=\delta^{2}\eta^{*}{}^{-1/3}\gamma_{c}$$

**Figure 6:**
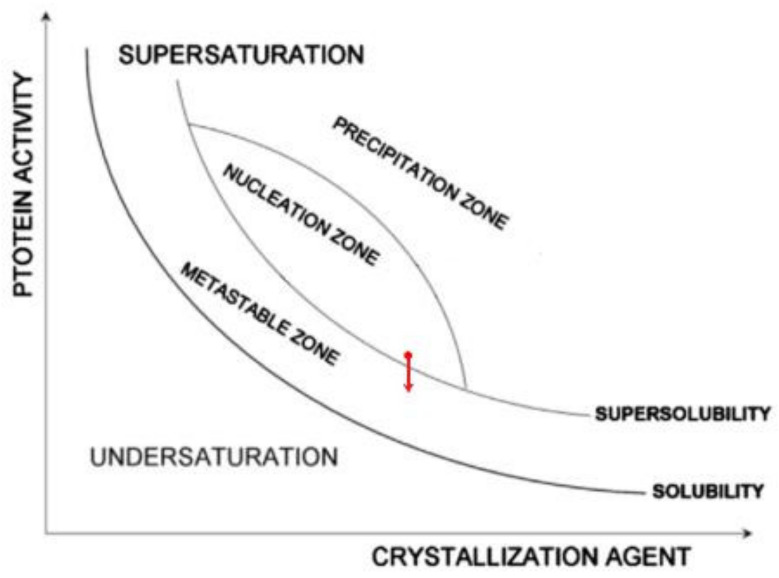
Ostwald–Miers phase diagram [17] illustrating the nucleation of a protein crystal in a pore (see the red point close to the super-solubility curve showing its presumable place in the nucleation zone where nucleation occurs), while, most probably, the crystal grows outside the pore orifice in the metastable zone, which is shown by the red arrow.

Comparing Equation (9) with Equation (11), we see that Δ*μ*_p_ is six times lower than Δ*μ*_v_. In other words, the unimpeded growth outside the pore orifice of the cubic crystal face (shown in Figure 4) of size $\delta^{2}\eta^{\frac{2}{3}}$ is secured by supersaturation equal to Δ*μ*_p_. Recalling that protein crystals nucleate in pores at moderate supersaturations (which are achievable in pores due to the synergistic diffusion–adsorption effect that arises from pore space confinement and interaction with pore walls), we suggest that the protein crystal nucleation in pores takes place in the nucleation zone, but close to the super-solubility curve (see the red point in Figure 6). Consequently, it is most likely that Δ*μ*_p_ is below the super-solubility curve, i.e., already in the metastable zone (see the red arrow in Figure 6).

**Figure 7:**
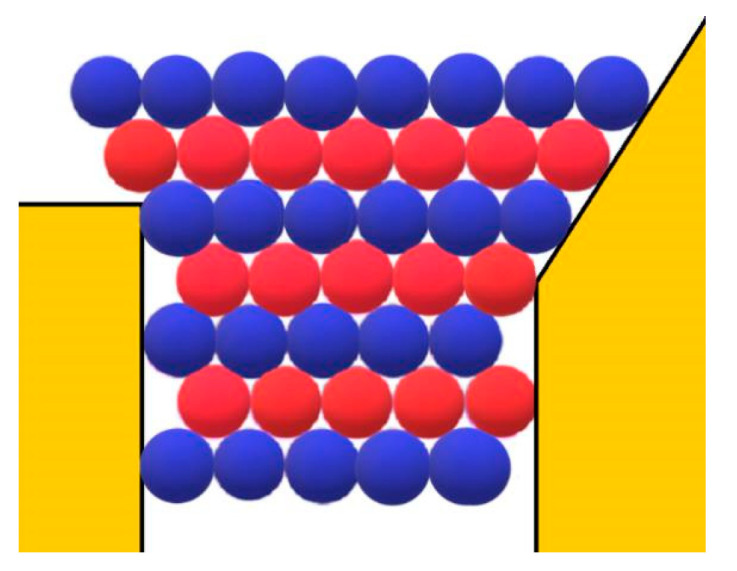
HCP protein crystal growing outside a pore with an inclined surface, shown on the right side of the pore. The adsorption of protein molecules on this surface facilitates the growth of the next crystal layer.

However, besides the stabilization of the crystal face at the pore orifice, which results from the protection of a considerable part of the crystal surface from the destructive action of water molecules, there are additional factors which promote its unimpeded growth. Apart from the open space outside the pore, another factor enabling the growth of the crystal outside it can be the adsorption of protein molecules on the surface of porous material. The shape of the pore orifice plays an important role in this respect: While angular pore openings (assumed by Page and Sear [18] and seen in Figure 3) hardly promote the adsorption of protein molecules, real ones (such as the one shown in Figure 7) that are generally devoid of sharp angles are more suitable for protein adsorption. Performing a random walk, some protein molecules arrive at the crystal face, which is level with the pore opening (see the two red balls at both sides of the pore orifice, approaching as shown by the arrows in Figure 3). However, they are not attached firmly enough to enable the lateral growth of the crystal outside the pore (the molecules in Figure 3 are attached to only two molecules of the crystal; for the closest-packed crystals especially, this is insufficient). More suitable in this respect are pore openings such as the one shown in Figure 7; the reason being that, in contrast to the case shown in Figure 3, the molecules on the right side of the crystal in Figure 7 also interact with the inclined surface of the pore opening itself. Thus, instead of only two intermolecular connections of energy *ψ*_b_, the impinging molecules bind firmly enough. The connection energy *E*_b_ of the peripheral row of length *λ* is:

(12) $$E_{b}=\lambda \left(2\psi_{b}+\psi \right)$$

where *ψ* is the adhesion energy of a single protein molecule to the pore material.

Although neglected in [18], the adhesion energy *ψ* is a significant factor contributing to the unimpeded growth of the protein crystal outside the pore; because larger molecules can contact the solid surface at more sites, the adsorption of protein molecules at such surfaces is strong even when affinity is very moderate [19]. In conclusion, the protein crystal grows outside the pore orifice without the need for 3D nucleation.

### 2.3. Experimental: Diffraction Quality of Protein Crystals Grown by Using Porous Materials

It is well known that the nucleation of crystals requires much higher supersaturation than is sufficient for their growth. The initial high supersaturation will, however, cause a rapid growth of crystals in which case, as already mentioned, the crystals will incorporate a larger number of impurities, which in turn cause defects. Persisting in the grown crystals, these defects deteriorate their diffraction quality. Hence, to grow more perfect crystals, the time of rapid growth must be shortened as much as possible. With that end in view, the stages of crystal nucleation and growth are sometimes separated, and the high supersaturation that was needed for the initiation of crystal nucleation is lowered during the subsequent growth stage. Usually, such an experimental approach yields better crystals. However, the choice of the exact moment at which to lower the supersaturation depends on the experimenter’s experience, and/or is determined by trial and error.

The important practical advantage of porous nucleants is that by using them, the separation of the nucleation and growth stages becomes superfluous. The reason for this is that only protein concentrations in the metastable zone are applied for growing protein crystals, i.e., the supersaturation is already lowered before inserting the porous nucleants. (Indeed, nucleants are only added in conditions that are known from preliminary experiments to provide insufficient supersaturation to yield crystals). In contrast, supersaturations above the super-solubility curve, which are necessary for the additional nucleation of 3D crystals outside the pore, are never used.

Our data for the actual diffraction of proteins on heterogeneous solid nucleants are reported here. Another porous material has been successfully tested as a protein crystal nucleant: molecularly imprinted polymers (MIPs). It should, however, be noted that the pores in the MIPs are too small (of the size of one, or possibly very few, protein molecules); therefore, no growth inside such pores is possible. Thus, this part of the theory does not apply to the MIPs, which function via a different mechanism of specific molecular affinity. However, the MIPs do decrease the supersaturation that is needed for protein crystal nucleation. Thus, the protein crystals grow under a lower supersaturation, and according to the theoretical expectation, they should be of a better diffraction quality. This theoretical expectation has also been confirmed by our experiments with MIPs, where six out of eight proteins tested under metastable versus higher supersaturation conditions (which were otherwise identical) yielded better diffracting crystals [8,10].

Diffraction data have previously been obtained for three target macromolecules (two proteins and one modified cyclodextrin), all of which required improved diffraction in order to determine their structure (Table 2).

1. Crystals of the C1 domain of the human cardiac myosin-binding protein-C (MyBP-C), obtained on buckypaper (made from an aggregate of single-walled carbon nanotubes and surfactant Triton X-100), diffracted to a maximum resolution of 1.6 Å (more typically in the 2.0–2.2 Å range). This is far superior to the best crystals obtained using standard techniques, which only diffracted to 3.0 Å [7]. The dominant pore size in the buckypaper was 9 nm. The crystals grew at metastable conditions in the trials with porous material (10 mg/mL protein in 50 mM NaCl and 20 mM Tris pH 7.0, equilibrated by vapor diffusion against a reservoir solution of 18% polyethylene glycol (PEG) of mean MW 3350 and HEPES buffer, pH 7.3). The conventionally grown crystals were obtained from 20% PEG reservoir solutions [7]. Importantly, in all cases, the crystals in the drops containing nucleant were single, i.e., not in clusters that may have appeared if repeated nucleation of novel 3D crystals outside the pore had occurred.
2. Crystals of InHr2 were obtained in the presence of bioglass at metastable conditions as well as at ‘borderline metastable’ conditions, i.e., conditions that gave visible crystals overnight in the presence of bioglass, but only after 6 days in the absence of porous material (10 mg/mL protein equilibrated against a reservoir solution of 11% PEG of mean MW 3350, 0.1 M imidazole at pH 7.0 and 75 mM MgCl_2_). The best of these crystals, obtained from the latter conditions, diffracted to 3.2 Å, in comparison to ca. 5 Å for routinely obtained crystals at higher PEG concentrations.
3. Crystals of the cyclodextrin derivative per(6-guanidino-6-deoxy)-γCD (gguan) that were obtained at metastable conditions in the presence of bioglass diffracted to better than 1.08 Å, whereas all X-rayed crystals routinely grown in standard, conventionally optimized conditions, diffracted to 1.3 Å at best. This improvement in resolution enabled the determination of this unusual structure [20].

Two model proteins were also crystallized with and without bioglass as part of the present study, using known crystallization conditions (see Section 4 and Table 2).

**Figure 8:**
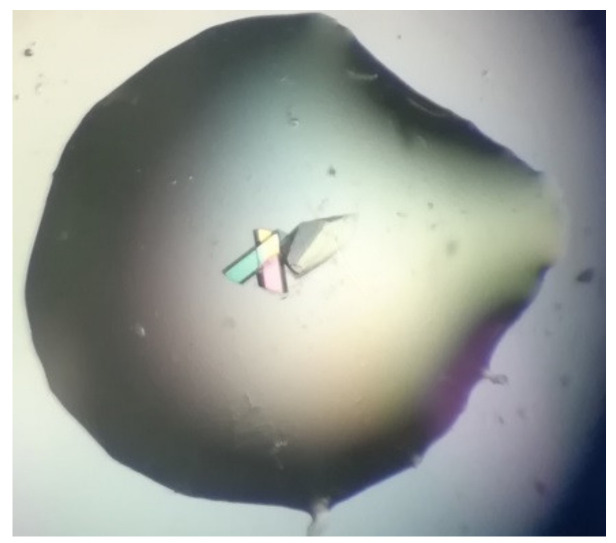
α-chymotrypsin crystals growing on bioglass at metastable conditions (drop volume after equilibration ca. 1 μL; crystals visible to the left of the bioglass grain).

**Figure 9:**
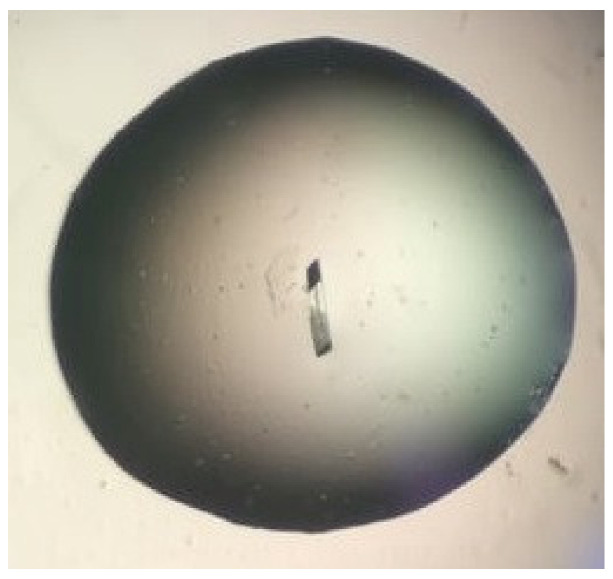
Ribonuclease A crystal growing on bioglass at metastable conditions (drop volume after equilibration ca. 1 μL; crystal visible to the right of the faintly visible bioglass grain).

1. Bovine α-chymotrypsin at metastable conditions (20 mg/mL protein in 25 mM NaOAc pH 4.8, equilibrated against several ammonium sulfate concentrations in the range 0.8–1.3 M ammonium sulfate in the same buffer) yielded medium-sized crystals in the presence of bioglass (and none in the controls). Most crystals were in contact with the bioglass grains (Figure 8). All trials (bioglass and controls) yielded much smaller crystals at higher supersaturations.
2. Bovine ribonuclease A at metastable conditions (12 mg/mL protein in 10 mM sodium citrate pH 5.0, equilibrated against 24–27% (*w*/*v*) PEG of mean MW 4000 and 50 mM of the same buffer) yielded small crystals in the presence of bioglass (and none in the controls). At a higher supersaturation (32% PEG), larger crystals of a very similar size and morphology were obtained both with and without bioglass, though the crystals grew appreciably faster in the presence of bioglass (Figure 9). The best crystal grown in the presence of bioglass diffracted to 2.8 Å versus 3.1 Å for the best crystal grown without bioglass.

To be completely convinced of the porous material’s ability to induce the formation of crystalline nuclei, we used controls in each crystallization trial. While crystals were observed in the samples containing porous nucleants, the controls set up under exactly the same conditions, but without addition of nucleant, were crystal-free. Undeniably, this is completely convincing evidence that the porous material acted as a nucleant.

## 3. Discussion

Using the Ising model, Page and Sear [18] considered nucleation and the growth of crystals in pores and outside them. They observed that, after the pore was filled, the crystallization process underwent a relatively long pause, and resumed later. The pause was attributed by the authors to the existence of a second nucleation barrier; thus, they suggested that 3D crystal nucleation is needed to continue the crystallization process. Page and Sear state that “the nucleation often proceeds via two steps: nucleation of pore filling, and nucleation out of the pore”. However, this conclusion may result from assumptions made by the authors for a sharply edged rectangular shape of the pore orifice, instead of the more realistic inclined shape shown in Figure 7, as well as from the absence of protein molecule adhesion to the surface of the porous material. Furthermore, the rigidity of the protein molecule must be considered. Lysozyme, for instance, possesses a highly stable structure, whereas ‘soft’ protein molecules can adapt to pores more easily [13]. Thus, different proteins prefer different pore sizes (hence, the most appropriate supersaturation levels can be case specific, and protein nucleation materials with pores of various widths, such as bioglass, are preferred).

In our approach, considering the protective role of the pore walls and the adsorption of the protein molecules on the surface of the porous material, we showed that when coming out of the pore, the crystal can grow unhindered. This means that a secondary nucleation is hardly indispensable for the growth of the crystal outside the pore. (Importantly, this thermodynamic result cannot be refuted by any non-thermodynamic method, including the method used in [18]). In conclusion, as a natural prolongation of the crystal in the pore (Figure 7), the protein crystal emerging from the pore orifice grows outside it without the need for 3D crystal nucleation.

Moreover, it is important to note that a secondary nucleation outside the pore (as suggested in Ref. [18]) may be detrimental to the quality of the grown crystal. It is likely that the nucleation process will not be restricted to a single 3D crystal; the formation of several crystal nuclei may also occur. This would, in turn, lead to the formation of sub-grains in the growing crystal, which would lead to a deterioration in its diffraction quality. In fact, it was established that such 2D crystal lattice defects can degrade the diffraction quality of protein crystals to a greater extent than one-dimensional defects, the so-called dislocations [21]. Koizumi et al. [21] have also shown that the large stress exerted on the sub-grains of protein crystals could potentially be controlled by introducing grown-in dislocations in the crystal. Importantly, it has been shown that dislocations can grow from seed crystals chemically cross-linked by glutaraldehyde [22].

Rejecting the necessity of 3D crystal nucleation at the pore opening, we underline that the diffraction quality of protein crystals grown using porous materials is improved, because the supersaturation under which these crystals nucleate and grow is lower compared to the one needed for conventional crystal nucleation [7]. Based on the observations in Refs. [21,22], a further working hypothesis is that the misfit between the crystal lattice and the supporting porous material can generate grown-in dislocations, leading to the disappearance of any local strain that may arise due to possible sub-grains, resulting in an improvement in diffraction quality of the protein crystals. Moreover, because the misfit between the crystal lattice and the supporting porous material can be greater than in the chemically cross-linked crystals, we suggest that grown-in dislocations appear more easily in the former case.

## 4. Materials and Methods

Hanging drop vapor diffusion trials were set up for two model proteins at and around known crystallization conditions, in the presence and absence of bioglass.

Bovine α-chymotrypsin (C3142, Sigma-Aldrich, Steinheim, Germany) was dissolved at 20 mg/mL in 25 mM sodium acetate pH 4.8. Vapor diffusion crystallization trials were set up under six conditions of varying precipitating agent concentrations, i.e., 0.8–1.5 M ammonium sulfate, in the same buffer. The trials were set up as sitting drops in a Linbro plate. A total of 1 μL protein stock solution was mixed with 1 μL from each precipitating agent reservoir solution (0.5 mL reservoir volume) and dispensed onto siliconized glass coverslips, which were then sealed over each well of the Linbro plate, using high-vacuum silicone grease, and left to equilibrate. The plate was incubated at 16 °C.

Bovine ribonuclease A (RNase A, Sigma, R5500) was dissolved at 12 mg/mL in 10 mM sodium citrate pH 5.0. Vapor diffusion crystallization trials were set up at six conditions of varying precipitating agent concentrations, in this case 24–35% (*w*/*v*) PEG of mean MW 4000, in 50 mM of the same buffer. The trials were set up as described above for α-chymotrypsin.

Bioglass grains were added with the help of tweezers or acupuncture-type needles in the crystallization drops, immediately before sealing the coverslip over the well. Each crystallization condition, in the presence and absence of bioglass, was set up in duplicate. Metastable conditions were defined as the ones where both drops without bioglass remained clear within the timescale of the experiment (ca. 3 weeks). 

## 5. Conclusions

Theoretical considerations of the growth of protein crystals in and outside pores of idealized shapes have shown why crystals grown with the aid of porous materials can have better X-ray diffraction quality compared to crystals grown under the same conditions, but in the absence of such substrates. Furthermore, the improved diffraction quality of some protein crystals grown on bioglass was confirmed experimentally. Importantly, it was argued that the crystal growing outside the pore (which is the part of the crystal that will eventually undergo diffraction), is a natural prolongation of the crystal nucleated and grown inside the pore. Growing under lower supersaturation compared to the one needed for conventional crystal nucleation and growth, such a protein crystal incorporates fewer impurities, thus having better diffraction quality.

## Figures and Tables

**Table 1:** Results for HCP crystal calculated by means of Equations (1) to (6). The analysis stops at *λ* = 6 because the tendency is clear, and the consideration of larger crystals is superfluous.

Thickness of Crystal Growing in Pores	Number of Molecules in the Edge of the Hexagonal Layer (*L* = *λ*)			
	3	4	5	6
	Crystal Binding Energy			
$\Delta G_{v}^{h}$ **(In 2D nucleus monolayer)**	42	90	156	240
$\Delta G_{v}^{h}$ +$\;\Delta G_{v}^{d}$ (in two layers) ^1^	102	234	420	660
$2\Delta G_{v}^{h}$ +$\;\Delta G_{v}^{d}$ (in three layers) ^2^	180	405	720	1125
$\mathrm{Ratios}\;(\mathrm{In}\;\psi_{b}$ $/\psi_{d}$ **)**				
$\;\Delta G_{v}^{h}/\Delta G_{s}^{h}$	2.21	2.43	2.56	2.64
($\Delta G_{v}^{h}$ +$\;\Delta G_{v}^{d}$)/$\Delta G_{s}^{d}$	8.5	8.67	8.75	8.8
($2\Delta G_{v}^{h}$ +$\;\Delta G_{v}^{d}$)$/\Delta G_{s}^{h}$	9.47	10.9	11.80	12.36

^1^ The bonds between the two layers are added. ^2^ The bonds between the three layers are added.

**Table 2:** Resolution limits of crystals grown with a porous nucleant in the metastable zone of conditions versus crystals grown in the absence of a nucleant in the spontaneous nucleation zone.

Protein	Resolution Limit in Presence of Nucleant at Metastable Conditions (Å)	Resolution Limit at Nucleation Conditions (Å)
C1 domain of MyBP-C	1.6 (more typically 2.0–2.2)	3.0
InHr2	3.2	5
gguan	1.08	1.3
RNase A (model protein)	2.8	3.1
